# Sleep Disorders in Infants and Toddlers with Hypoxic Ischemic Encephalopathy Treated with Therapeutic Hypothermia: A Case–Control Study Using the SDSC

**DOI:** 10.3390/children12081058

**Published:** 2025-08-12

**Authors:** Domenico M. Romeo, Chiara Arpaia, Maria Rosaria Lala, Giorgia Cordaro, Claudia Brogna, Marianna Moro, Francesca Gallini, Giovanni Vento, Eugenio Mercuri

**Affiliations:** 1 Pediatric Neurology Unit, Fondazione Policlinico A. Gemelli, IRCCS, L.go A. Gemelli 8, 00168 Rome, Italy; chiara.arpaia@guest.policlinicogemelli.it (C.A.); mariarosaria.lala01@icatt.it (M.R.L.); g.cordaro@ausl.mo.it (G.C.); claudia.brogna@policlinicogemelli.it (C.B.); marianna.moro@guest.policlinicogemelli.it (M.M.); eugeniomaria.mercuri@policlinicogemelli.it (E.M.); 2 Pediatric Neurology Unit, Università Cattolica del Sacro Cuore, 00168 Rome, Italy; 3 Neonatal Intensive Care Unit, Fondazione Policlinico Universitario A. Gemelli, IRCCS, L.go A. Gemelli 8, 00168 Rome, Italy; francesca.gallini@policlinicogemelli.it (F.G.); giovanni.vento@policlinicogemelli.it (G.V.)

**Keywords:** sleep disorders, asphyxia, hypothermia, infants, toddlers, SDSC

## Abstract

Background and Objectives: Sleep complaints are particularly relevant in the development of children, affecting cognitive development, neuropsychological functioning, and learning abilities. The aims of this study were as follows: (i) to determine the incidence of sleep disorders in low-risk infants and toddlers with hypoxic ischemic encephalopathy (HIE) treated with therapeutic hypothermia (TH), using the Italian version of the Sleep Disturbance Scale for Children (SDSC); and (ii) to compare the data with those of a healthy control group. Materials and Methods: This is a cross-sectional case–control study involving a total of 167 infants and toddlers (aged 6–36 months) with HIE treated with TH and 160 typically developing infants assessed using the SDSC filled out by the mother. A neurocognitive assessment was also performed. Exclusion criteria were mild perinatal asphyxia, major brain lesions, congenital malformations, severe postnatal infectious diseases, metabolic complications, cerebral palsy, neurodevelopmental impairment, and epilepsy. Results: In the study group, an abnormal total SDSC score was found in 1.8% of infants; 10% of infants had an abnormal score on at least one of the SDSC factors. No specific differences in the SDSC total and the factor scores were observed between the study and control group, with the exception of difficulties in maintaining sleep and sleep hyperhidrosis, with higher scores in HIE infants. Conclusions: Low-risk infants and toddlers with HIE showed a low incidence of sleep disorders, similar to those observed in control group, with some exceptions. As these incidences may increase significantly with age, further clinical assessments will be needed to confirm these data at older ages.

## 1. Introduction

Sleep disorders are a very frequent problem in infancy, especially for children at preschool age [1,2,3].

Current evidence supports the general recommendation that children aged 4 months to 18 years should regularly receive an adequate amount of sleep per 24 h to promote optimal health. Based on this evidence, the American Academy of Sleep Medicine have developed consensus recommendations for the amount of sleep needed to promote optimal health in children and adolescents [3].

Several studies have underlined the role of sleep in relation to behavioral, emotional, and social problems [4,5], showing that sleep characteristics represents a good predictor of neurodevelopmental outcome [4,5,6,7,8,9,10]. In the first stages of development, sleep has different characteristics than in later periods of human life, mainly due to qualitative and quantitative changes in the structuring of sleep. Adequate sleep is therefore essential to the brain development of children. Indeed, there are increasing correlations between the organization of sleep in early life and cognitive development, neuropsychological functioning, and learning abilities [8].

A recent study analyzed the frequency and prevalence of sleep disturbances using the Sleep Disturbance Scale for Children (SDSC) in a large Italian sample of children and adolescents with autistic spectrum disorder, reporting specific predictors of the presence of sleep disturbances. The study found an elevated global score for sleep disturbance in 33% of participants, with a prevalence of disturbance in initiating and maintaining sleep [10]. The SDSC [11] represents one of the most widely used questionnaires for sleep disorders in pediatric patients, even at early ages, and it has been validated in several studies with different populations of infants with neurodevelopmental disorders [9,10,11,12,13,14].

There are no studies in the literature that have systematically evaluated the incidence of sleep disorders using the SDSC in children with a history of asphyxia at birth treated with therapeutic hypothermia (TH). Neonatal hypoxic ischemic encephalopathy (HIE) is one of the major causes of newborn death and long-term disability in the world [15,16,17,18]. The present gold-standard treatment for moderate and severe neonatal HIE is TH, which significantly decreases, but does not eliminate, mortality and adverse neurodevelopmental sequelae [16,17].

Hypoxic ischemic insult may play a role in the primary brain injury and in the higher frequency of sleep disorders [15]. Some previous studies have underlined that in these patients, the presence and quality of sleep–wake cycling (SWC) reflected the severity of the injury, and a good SWC is associated with good neurodevelopment outcome [18].

In an observational study carried out in an Irish population with a history of perinatal asphyxia [19], it emerged that in the preschool age bracket, there is a significant percentage of patients who have quality of life problems and, more specifically, sleep problems.

Ding et al. [20] also described sleep disorders in preschool children with a history of hypoxic ischaemic encephalopathy. Their paper shows a higher incidence of problems in inducing and maintaining sleep in infants with severe HIE at birth. In cases of moderate HIE, respiratory disturbances during sleep and circadian rhythm alterations are prevalent. However, the link between neonatal asphyxia treated with TH and sleep disturbances is still not well understood. Furthermore, no specific study has been reported on younger infants with HIE; this could be of particular interest for clinicians in terms of early monitoring and management, as sleep disorders constitute a precursor to, and potential early indicator of psychopathology [9].

Our hypothesis is that infants with HIE at less than 3 years old could show a different incidence of sleep disorders than that observed in a control group of peer infants using a structured tool like the SDSC.

Therefore, the aims of this study were as follows: (i) to evaluate the incidence of sleep disorders, using the Italian version of the SDSC (6–36 months) [9], in a low-risk population of infants and toddlers with HIE treated with TH; and (ii) to compare the data with those of a healthy control group.

## 2. Materials and Methods

### 2.1. Population

This is a cross-sectional case–control study involving infants that was part of a prospective study of infants with HIE treated with TH followed regularly at the Paediatric Neurology Unit of the Fondazione Policlinico Universitario A. Gemelli in Rome between January 2020 and December 2022.

For the purpose of this study, infants ≥ 36 weeks gestation with signs of encephalopathy > 10 min after birth but before 6 h were recruited if they had one of the following signs: (i) a pH ≤ 7.0 or a base deficit ≥ 16 mmol/L in the first hour of life on cord or infant arterial blood; or (ii) an abnormal intra-partum course and either a 10 min Apgar score ≤ 5 or continued respiratory support at 10 min [21]. Infants were candidates for the study when encephalopathy or seizures were present. The encephalopathy was graded as mild, moderate, or Severe according to clinical signs previously published [21,22]. These assessments were performed within 1 h. All the infants who met the inclusion criteria and had moderate or severe encephalopathy underwent TH according to the international guideline for 72 h [21,22].

Exclusion criteria were mild perinatal asphyxia, major brain lesions observed on US scans, congenita malformations, severe postnatal infectious diseases, metabolic complications, neurodevelopmental disability, cerebral palsy (CP), and epilepsy.

### 2.2. Sleep

Data related to sleep disturbances were collected using the 19-item Italian version of the Sleep Disturbance Scale for Children (SDSC) for Infants and Toddlers validated for infants between 6 and 36 months [9].

This scale is composed by 19 items according to the Likert scale values of 1–5, where a higher numerical value reflects a higher clinical severity of symptoms (1 = never; 2 = up to once or twice per month; 3 = once or twice per week; 4 = 3–5 times per week; 5 = daily).

This scale analyzes most of the areas of sleep disorders in preschool children: disorders in initiating sleep (DIS), related to problems in falling asleep; difficult in maintaining sleep (DMS), related to awakening during the night; sleep-disordered breathing (SBD); parasomnias (PAR), which includes nightmares, sleepwalking, and sleep terrors; sleep hyperhidrosis (SHY); and disorders of excessive somnolence (DOES), related to daytime somnolence.

The total sleep score and the sum of single factors were transformed in a T-score by referring to the scoring sheet validated on a normative sample and based on the standard formula [T-score = 50 + (Value − Mean)/Standard Deviation × 10].

A T-score of more than 70 was considered abnormal, while a T-score between 61 and 70 was considered a “borderline” score.

This questionnaire was dispensed to the primary caregiver of the infants, who was the mother in all cases, during the routine neurological assessment in our Unit.

The SDSC was further dispensed to the primary caregivers of a control group population of healthy peers recruited via nurseries. The mothers filled out the questionnaires during school hours under the control of the researchers who distributed the questionnaires, and no missing values were reported. Infants with neurodevelopmental disabilities or receiving ongoing prescription drugs (benzodiazepine, melatonin, antihistaminic drugs, and antiepileptic drugs) were excluded as all these factors could negatively impact the development of sleep and could therefore cause a population bias.

### 2.3. Neurodevelopment Assessments

According to our clinical routine follow-up, all the infants performed a neurological examination using the Hammersmith Infant Neurological Examination (HINE) at 12 months [23] and a neurodevelopmental assessments at 24 months of age using the Griffith’s Mental Development Scales [24]. The study protocol was approved by the Ethics Committee of our Institution (ID: 3544, prot. N. 0051769/20; date: 21 December 2020), and informed consent was obtained from parents.

### 2.4. Statistical Analysis

Data were presented as the mean values and range for continuous variables normally distributed, and as count and percentages for categorical variables. The Shapiro–Wilk test and Shapiro–Francia test were applied to the sample to verify a non-normal distribution. The comparison between infants with HIE and the control group for the SDSC total and 6 factor scores and age were performed using the non-parametric Mann–Whitney U test. A 2-tailed value of *p* < 0.05 was considered significant. Subcategory analysis were performed using the previously described sleep categories.

The sample size (*n* = 167) was calculated considering the number of low-risk HIE infants treated with TH in our region during the study period (*n* = 295), the margin of error (5%), and the confidence level (95%).

## 3. Results

During the study period, 167 low-risk infants with HIE treated with TH (103 male and 64 female) with a mean age of 18.4 months (range: 6–36) fulfilled the inclusion criteria (Figure S1). All the infants reported a score at the Griffith’s Mental Development Scales and a neurological assessment within the normal range.

The SDSC was filled out by the mothers of the infants at a mean age of 18.5 months (range: 6–36 months).

The questionnaire was also completed by the mothers of 160 typically developing infants (72 male; 78 female) with a median age of 20 months (range: 6–36 months). This control group presented the same age and gender distribution of the study group.

### 3.1. SDSC Results in Infants with HIE

An abnormal total sleep score (>70) was found in 3/163 (1.8%) infants in the group with HIE treated with therapeutic hypothermia. In this group, a borderline score (T-score between 61 and 70) was found in seven infants (4.2%). A total of 17 children (10.4%) had an abnormal score on at least one of the SDSC factors. Specifically, the analysis revealed three (1.9%) infants with significant scores on the DIS scale, two (1.9%) on the DMS scale, four (2.4%) on the SBD scale, six (3.6%) on the PAR scale, two (1.2%) on the DOES scale, and eight (4.9%) on the SHY scale.

### 3.2. SDSC Results in Control Group

An abnormal total sleep score (>70) was found in 3/150 term-born infants (2%), and a total of 13 infants (8.6%) had an abnormal score on at least one of the SDSC factors.

Specifically, the analysis revealed two (1.3%) infants with significant scores on the DIS scale, none (0%) on the DMS scale, four (2.6%) on the SBD scale, six (4.0%) on the PAR scale, one (0.6%) on the DOES scale, and one (0.6%) on the SHY scale.

### 3.3. Comparison of Sleep Disorders Between Infants with HIE and Control Group

No statistically significant differences between the two groups were observed in the SDSC total score and in the DIS, SBD, PAR, and DOES scores (*p* > 0.05). HIE infants reported higher significant scores in DMS and SHY (*p* < 0.01) (Table 1).

## 4. Discussion

The outcomes resulting from perinatal hypoxic ischaemia can be relevant variables and include different degrees of brain and motor function impairment. Therapeutic hypothermia improved the outcome of infants with moderate-to-severe HIE, mainly in increasing survival without neurological abnormalities at a follow-up of 6–7 years of age [15,16,17]. Sleep disturbances are frequently reported in patients diagnosed with HIE and concomitant motor impairment, such as CP [18]. However, it is noteworthy that sleep disturbances are also documented in non-severe hypoxic ischemic patients. For example, Ding et al. [20] investigated the sleep qualities of children at preschool age, with different degrees of HIE: moderate HIE was significantly associated with sleep initiation/maintenance difficulties and related breathing problems, whereas mild HIE was significantly associated with circadian rhythm alterations. A similar set of results was observed in a study evaluating quality of life and the presence of sleep disturbances in school-age children following neonatal encephalopathy (NE) [17]. This study found significantly higher sleep disorders in post-NE children compared with controls, with greater bedtime resistance and sleep anxiety observed in the post-NE group.

The present study is the first to assess sleep disorders in 6–36-month-old infants with HIE using the SDSC. An abnormal total sleep score was reported in 1.8% of the population, with an abnormal score on at least one SDSC factor in 10.4%. No statistical differences were observed between HIE infants and control group in terms of SDSC total scores and in most of the other factors, with the exception of DMS and SHY. Our hypothesis is that at early ages, all criticalities linked to low-risk HIE infants may not yet have emerged in all their complexity and variability as they may manifest in more advanced stages of neurodevelopment, such as during school age. This hypothesis could be substantiated by previous studies that also identified a higher prevalence of behavioral problems at school age in subjects with a documented history of HIE [25,26,27].

Preschool children experience significant changes in the quantity and quality of sleep and their sleep–wake rhythm, which may lead to a higher prevalence of sleep disorders in this age group. Different studies show that preschool and school age children have the highest incidences of emotional problems and neurodevelopmental disorders, such as attention deficit/hyperactivity disorder (ADHD). Such difficulties are very common in children with difficulties at birth and often correlate with poor sleep quality, as documented by previous studies in the literature [28,29,30,31,32,33,34,35].

Another confirmation of our hypothesis is provided by a previous study of our research group performed in a population of low-risk preterm infants at early ages (6–36 months) [13]. In that study, an abnormal total sleep score was found in 1% of the preterm group, with an abnormal score on at least one SDSC factor in 16%, similar to those observed in the present study. The occurrence of global sleep disorders in preterm children was comparable to those reported in the control group in both the SDSC total and the factor scores. At older ages, higher significant median scores for sleep disorders in preterm children were observed compared with the control group using the same tool, along with an increase in behavioral problems [11].

Furthermore, in our sample, no infants with CP or other neurodevelopmental disorders were included, impacting the potential incidence of sleep disorders in the population.

The outcomes of HIE infants who received TH have significantly improved in relation to survival and neurodevelopmental disorders, including CP, yet some cognitive and/or behavioral impairment continues to occur and remain significant at preschool and school age, as shown by several studies [16,24,29,30,31,32,33,34,35]. For example, previous studies assessed outcomes following HIE in survivors without CP, finding difficulties in cognition and behavior, especially in older age groups, which were not always evident at a young age [24,26]. Furthermore, in these children, the total sleep scores were significantly higher than those in the control group, confirming that sleep disorders were evident at older ages, which is probably related to the improving of behavioral or cognitive problems during these periods [26].

However, in the present study, two SDSC factors (DMS and SHY) were significantly higher in HIE infants than in the control group. Sleep–wake cycling (SWC) in newborns with HIE often had a more discontinuous character compared with SWC in healthy newborns, even in those with normal outcomes, with an increased proportion of quiet sleep [18]. Therefore, the higher incidence of DMS reported in the present study could be related to this early different quality of SWC, which is also reported at older ages [18]. Furthermore, infants with HIE treated with TH reported a high incidence of externalizing behavioral problems, even at early ages, in the absence of motor or cognitive problems [36]. Behavioral problems in children are well known to be related to sleep disorders such as DIMS, Sleep/Wake Transition Disorders, and SHY [37]. In the present study, a behavioral assessment was not performed; therefore, we can only hypothesize with respect to the relationship between sleep disorders and behavior.

The present study has some limitations. First, the criterion used for measuring sleep disorders using clinical questionnaire is not objective and may be influenced by the different perceptions of various caregivers regarding the observed issues. The management of children with HIE could influence their perception of sleep disorders; in fact, on the one hand, as clinical caregivers, they may experience higher levels of professional support and specialized training; but on the other hand, they face greater pressure related to the specific needs of the baby and institutional protocols. More objective methodologies like actigraphy and polysomnography could eliminate such biases related to personal perception; however, these instruments could be poorly tolerated in children and not always feasible in clinical routines; moreover, parental questionnaires like the SDSC have the advantage of saving time and costs and can be administered at each outpatient assessment.

An additional limitation is represented by the absence of a structured examination of infants’ behavior that could be useful in relation to sleep disorders. In fact, sleep disturbances can greatly affect daytime behavior in children, leading to neurobehavioral disturbances such as both internalizing (emotional dysfunction, anxiety) and externalizing behaviors (inattention, hyperactivity), with a bi-directional relationship, probably due to a cortisol-level modification and genetic, environmental, and familial factors [38].

However, despite these limitations, we believe that our study contributes to furthering our understanding of sleep difficulties in HIE infants.

## 5. Conclusions

In conclusion, our results suggest that low-risk HIE infants and toddlers do not show differences in the incidence of sleep disturbance compared with control peers, with the exception of DIM and SHY. However, the literature suggests that these incidences may increase significantly during school age, with improvements in the behavioral symptoms in these patients. To confirm this data, a study is ongoing in the same population of infants,” assessing SDSC at an older age. These data could be useful for clinicians in screening those HIE infants at risk of sleep disorders. Identifying and treating sleep problems is particularly important because the occurrence of sleep problems is significantly related to lower quality of life and behavior in childhood and adolescence in HIE patients [39].

Screening for sleep disorders should be part of the regular-intake procedures for these children, even at early ages [38]. Consequent identification of a sleep problem, either as a maintaining or initiating feature of behavioral problems, can then be appropriately managed, counselling families in promoting good sleep hygiene, involving both a sleep-promoting environment and a bedtime routine.

Future longitudinal studies could focus on evaluating the same cohort at older ages to assess sleep patterns using consistent methodologies.

## Figures and Tables

**Table 1:** SDSC scores in children with perinatal asphyxia treated with therapeutic hypothermia and in control group.

	DIS	DMS	SBD	PAR	DOES	SHY	T SCORE TOTAL
**HIE** **Group**	45.7 ± 8.1	48.5 ± 8.2	47.3± 8.7	47.3 ± 9.4	46.1 ± 6.6	50.0 ± 10.2	45.8 ± 8.4
**Control** **Group**	46.5 ± 7.5	45.8 ± 6.9	47.7± 8.4	48.4 ± 8.4	47.7± 7.7	46.7 ± 5.8	46.0 ± 6.8
***p***	0.323	0.002	0.664	0.255	0.052	0.001	0.808

(DIS) disorders in initiating sleep; (DMS) difficult in maintaining sleep; (SBD) sleep-disordered breathing; (PAR) parasomnias; (DOES) disorders of excessive somnolence factor; (SHY) sleep hyperhidrosis.

## Data Availability

On request.

## Supplementary Materials

The following supporting information can be downloaded at: https://www.mdpi.com/article/10.3390/children12081058/s1, Figure S1: Flow-chart Population Follow-up.
