# Supplementary figures and images for: Sleep Disorders in Infants and Toddlers with Hypoxic Ischemic Encephalopathy Treated with Therapeutic Hypothermia: A Case–Control Study Using the SDSC
Source: Children (Basel). 2025 Aug 12;12(8):1058. doi: 10.3390/children12081058 (PMC12384304; doi:10.3390/children12081058)

Figure S1 Flow-chart Population Follow-up

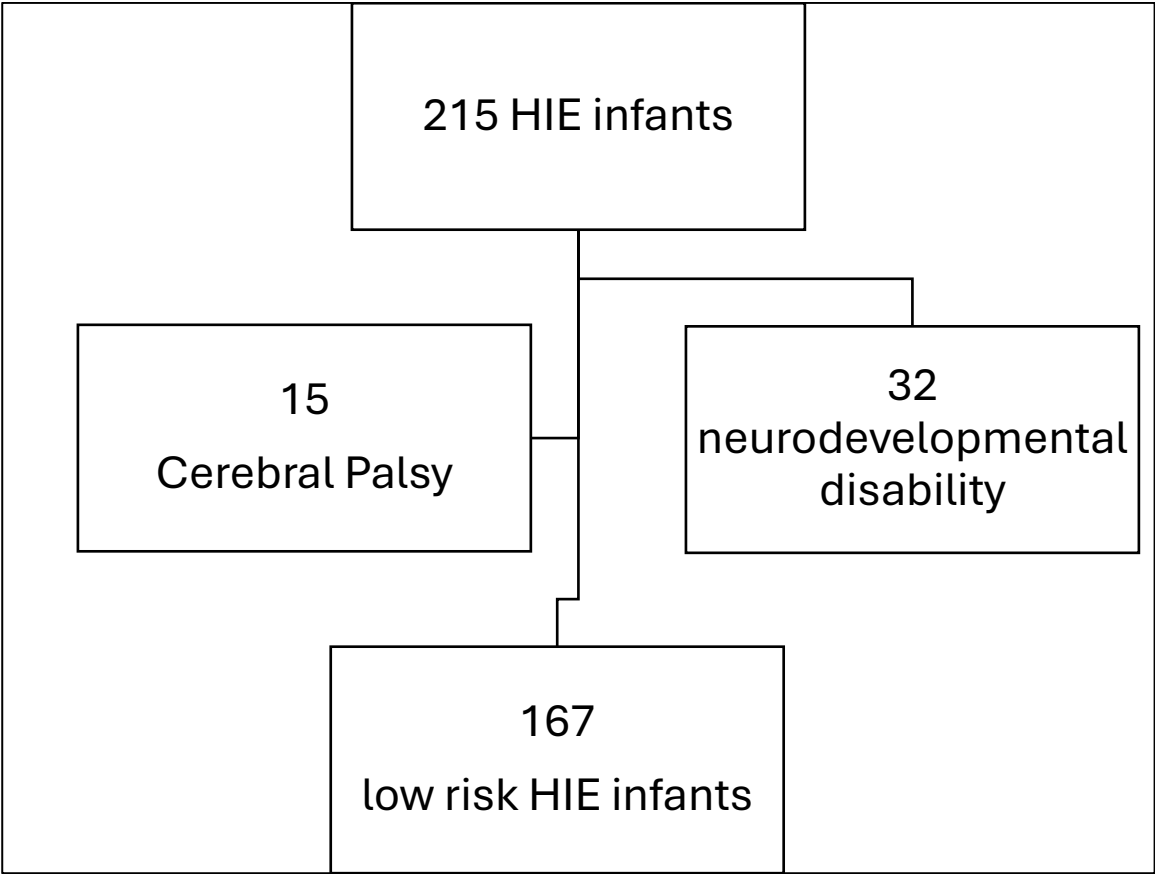

Supplement: Supplementary file 1 [file children-12-01058-s001.zip › children-3808289 Figure S1.pdf]
